# Supplementary material for: Microbial Synthesis of Lactic Acid from Cotton Stalk for Polylactic Acid Production
Source: Microorganisms. 2023 Jul 28;11(8):1931. doi: 10.3390/microorganisms11081931 (PMC10458930; doi:10.3390/microorganisms11081931)
Supplement: Supplementary file 1 [file microorganisms-11-01931-s001.zip › microorganisms-2493862-supplementary.pdf]

microorganisms

Supplementary Materials

Microbial Synthesis of Lactic Acid from Cotton Stalk for Polylactic Acid Production

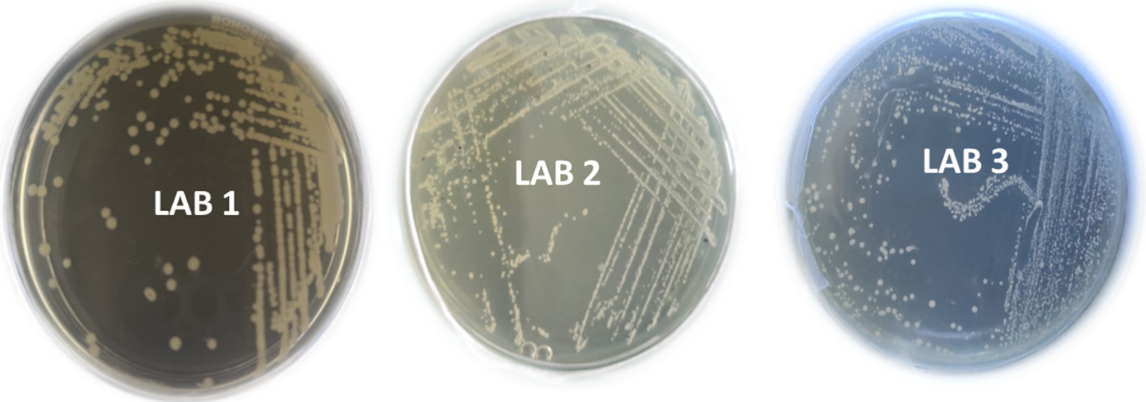

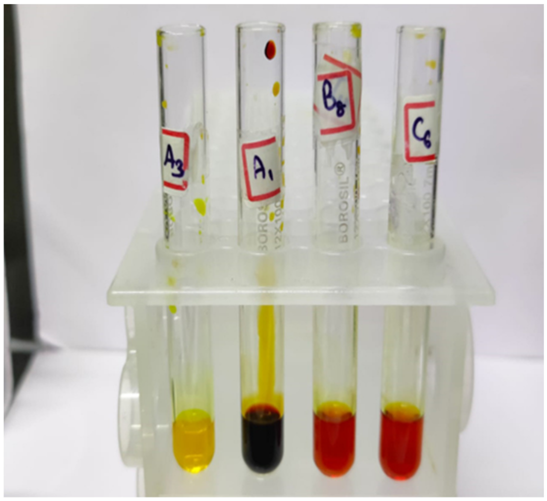

Glucose estimation using DNSA method of fermentation broth

Supplementary Figure S1. Isolation of lactic acid bacteria.

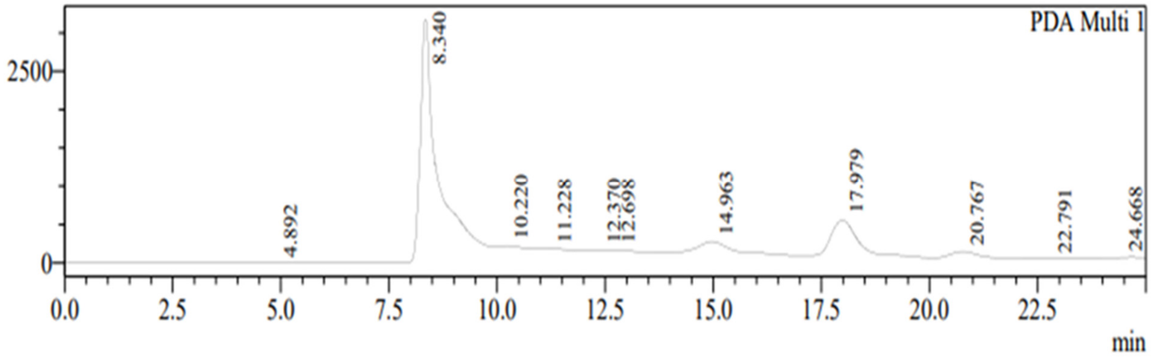

HPLC Chromatogram of LAB-2 isolate

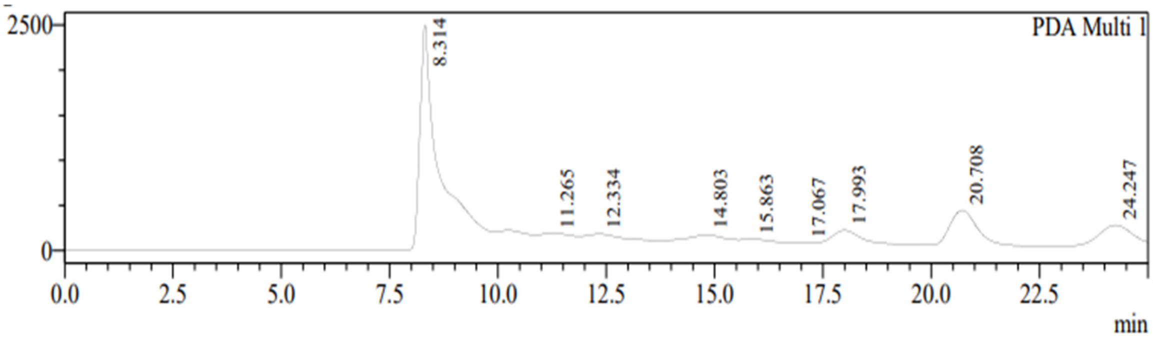

HPLC Chromatogram of LAB-3 isolate

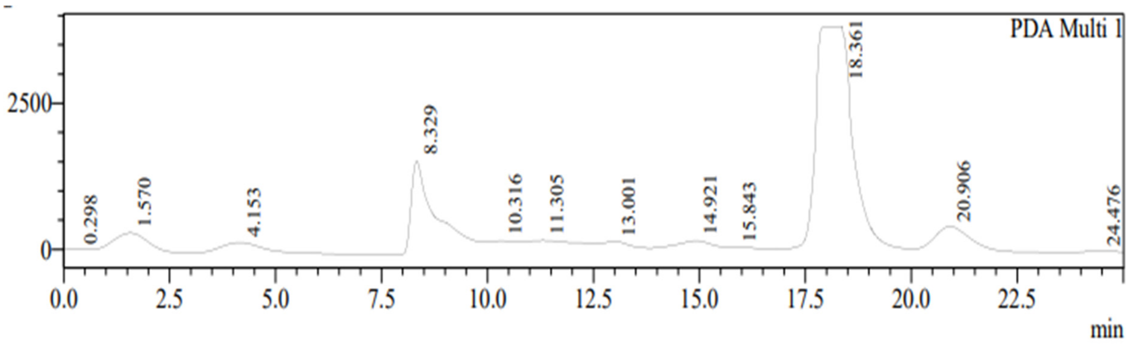

HPLC Chromatogram of LAB-4 isolate

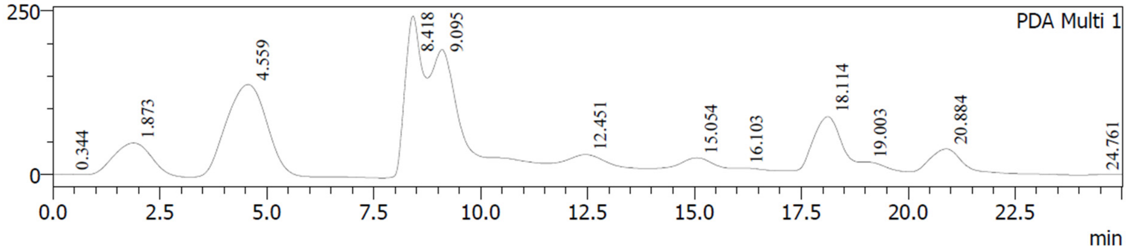

HPLC Chromatogram of LAB-5 isolate

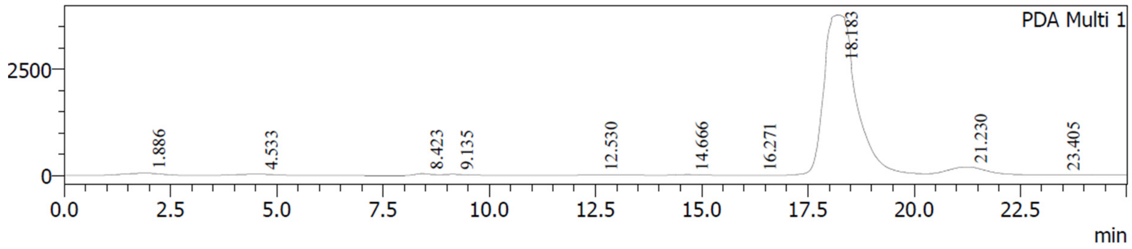

HPLC Chromatogram of LAB-6 isolate

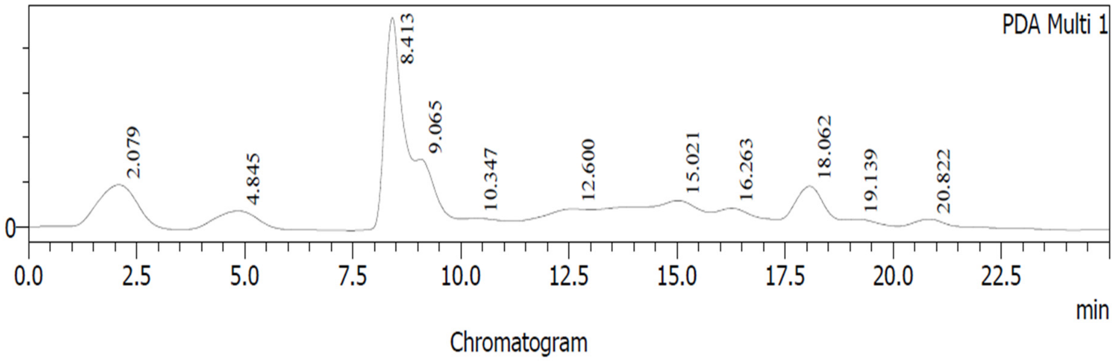

HPLC Chromatogram of LAB-11 isolate

Supplementary Figure S2. HPLC profiles of lactic acid concentrations by isolates.

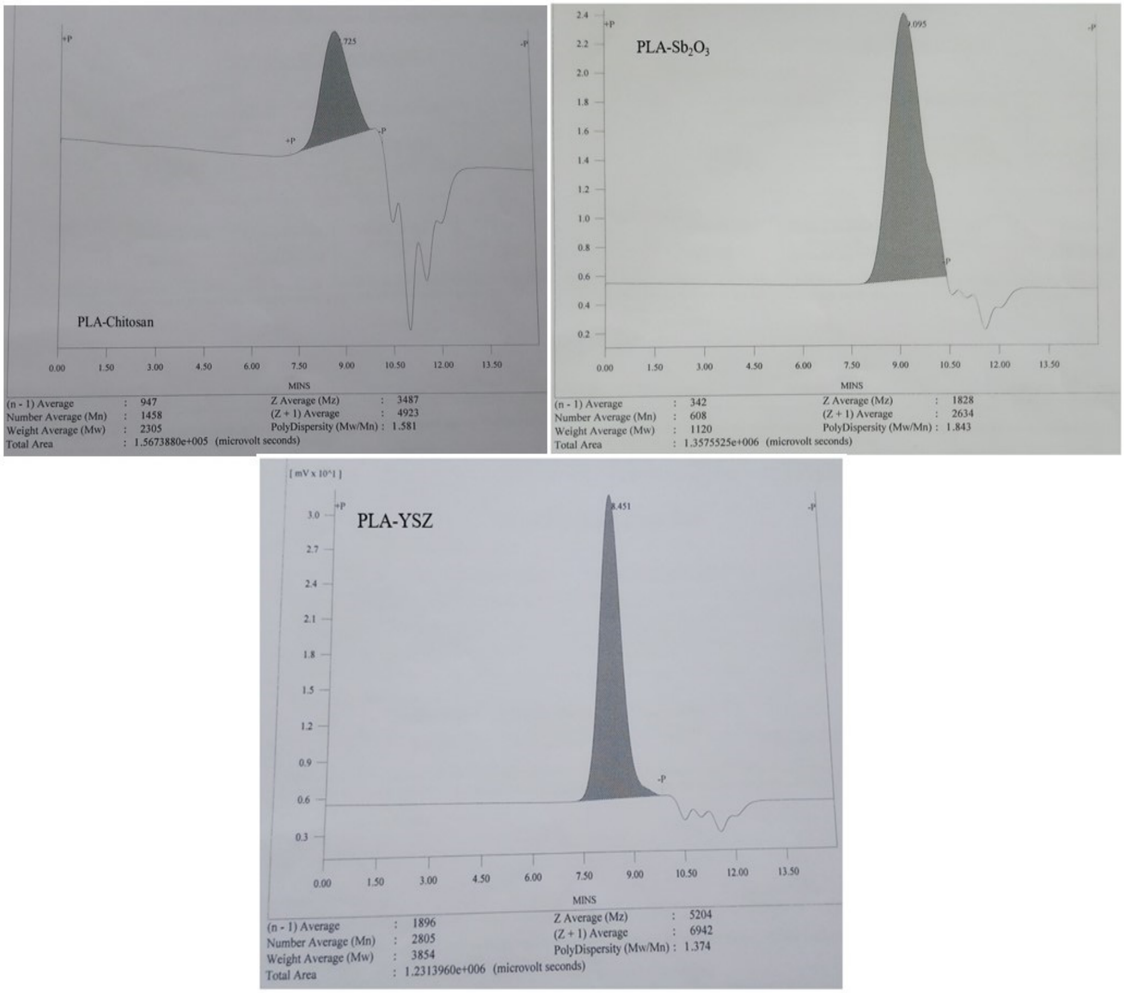

Supplementary Figure S3. GPC spectrum of resultant polymers i.e., PLA-Chitosan, PLA-Sb_2O_3, and PLA-YSZ.
